# Patient-Reported Outcome Measures in a Facial Reconstruction Case Series Following the Implementation of an Integrated Craniofacial Multidisciplinary Team Clinic, Three-Dimensional Photography, and Computer Modeling

**DOI:** 10.1093/asjof/ojad082

**Published:** 2023-09-20

**Authors:** Prateush Singh, Kayen Chan, Shivani Dhar, Imogen Ashby, Eva Krumhuber, Afshin Mosahebi, Allan Ponniah

## Abstract

**Background:**

Facial reconstruction surgery is often a complex and staged process, leading to lengthy reconstructive journeys for patients. The integration of a clinical pathway can give patients a clearer understanding of what to expect at each stage of their reconstructive journey.

**Objectives:**

The authors demonstrate how the incorporation of multidisciplinary team clinics, three-dimensional (3D) photography, and 3D modeling into an integrated pathway can streamline the process for patients undergoing facial reconstructive surgeries and aid their understanding of their surgeries.

**Methods:**

A novel clinical pathway was developed for patients undergoing facial reconstructive surgery at a tertiary reconstructive unit in London. A case series was collated of 35 patients who had been through the integrated pathway. Patient-reported outcome measures (PROMs) were assessed using FACE-Q scales, Global Aesthetic Improvement Scale, Self-Perception of Age score, and Ordinal Rank change in facial aesthetic appearance, determined subjectively and objectively. Statistical analysis was performed to calculate mean averages for each scale and PROM.

**Results:**

High patient satisfaction with overall facial appearance, aging appearance, and the decision-making process was demonstrated. The average perceived improvement in age-related facial appearance was −7.7 years postreconstruction compared with prereconstruction. The Ordinal Rank improvement on facial aesthetic appearance showed considerable improvement, both subjectively and objectively.

**Conclusions:**

The authors advocate the implementation of an integrated clinical pathway for facial reconstruction, with positive impacts observed in terms of patient satisfaction and objective assessments of facial appearance. Similar principles can be extrapolated to other aspects of reconstructive surgery.

**Level of Evidence: 3:**

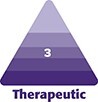

A major workload in plastic and reconstructive surgery arises from the need for facial reconstruction—after trauma, cancer, or congenital abnormality. The complexity and need for often staged reconstructions can lead to the formation of lengthy reconstructive journeys for patients, most of whom do not have a clear understanding of exactly what to expect at each stage. The skills of reconstructive surgeons lie in their abilities to discuss complex cases in multidisciplinary team (MDT) formats, collaborate with allied experts, explain accurately their reconstructive plan, and communicate these to patients, work in collaboration with patients to ensure that their expectations and needs are optimized, minimize patient anxiety, and ultimately produce functional and aesthetic reconstructions. This multidisciplinary format is similar to that of “cleft clinics” in the United States, where clinicians from different specialties meet regularly to formulate collaborative plans for patients. In order to streamline this process, we developed a novel clinical pathway for patients needing facial reconstruction at a tertiary reconstructive unit in central London. We combined MDT discussions with patient clinics, allowing them to have a 360° understanding of all aspects of their upcoming reconstructive journey and who would be involved and at what stage. Prior to the clinic, patients underwent 3-dimensional (3D) photography with images utilized in the MDT clinic. Computer models were then produced of expected ideal outcomes and the stages needed to get there. A computer database of patients who had undergone similar staged reconstructions was also shared to improve patient understanding and expectations at each stage. All these factors were designed to improve not only the efficiency of the reconstructions but also the quality of the patient's journey from their own perspective. In order to validate this, we carried out validated assessment questionnaires after their reconstructions.

To ensure that the reconstructions we were producing were both aesthetic and functional, we also carried out subjective patient-based assessments of their own facial appearance and had a panel of 10 independent plastic surgeon reviewers carry out the same assessment, thereby introducing an element of objectivity in our assessments. Most reconstructions are carried out by the surgeon and their patient in close communication, but often the patient is unable to truly comprehend the entirety of the plans of their surgeon. The aim of this study was to validate the usefulness of adding these elements into our facial reconstruction pathway and hence evaluate the use of such a pathway in other areas of reconstructive surgery.

## METHODS

A novel integrated craniofacial pathway was created at a tertiary plastic surgery center. This included an MDT clinic that consisted of consultants from various specialties (such as plastic surgery, maxillofacial, otolaryngology, and neurosurgery), and speech and language therapists. The study was conducted between October 2020 and April 2023. Patients presented for 3D photography, underwent an MDT clinic review, and then subsequently had 3D models developed for reconstructive surgical plans at each stage of their reconstructive journey as well as follow-ups postoperatively. All patients were given information in the clinic regarding what to expect in terms of surgical scars. They were informed that scars heal unpredictably and could take 12 months to mature, but that ultimately they should fade to acceptable limits. Patients were also advised to massage their scars at appropriate times, to help minimize the appearance of the scars. Patients who had been through this pathway with completed reconstructions then completed patient-reported outcome measures (PROMs) using 5 FACE-Q scales, Global Aesthetic Improvement Scale (GAIS), Self-Perception of Age score (Appendix A), and Ordinal Rank change in facial appearance attractiveness as determined subjectively by the patient and peer-reviewed by 10 independent reviewers (Appendix B). The questionnaires and peer-reviewed analyses were performed at complete follow-up postreconstruction.

The 5 FACE-Q scales are a 10-item satisfaction with facial appearance overall scale, a 6-item satisfaction with outcome scale, a 6-item satisfaction with decision scale, a 7-item aging appearance appraisal scale, and an 8-item appearance-related psychological distress scale. Scores were collated and Rasch transformations performed to obtain scores from 0 to 100.

The Self-Perception of Age score was calculated by asking patients to detail their actual age, and then their perceived age after surgery—with positive values indicating that they felt older and negative values that they felt that number of years younger. The GAIS utilized a 5-point subjective self-rating scale from −2 to +2, with patients asked to indicate how they felt that their global aesthetic appearance had changed postoperatively, with 2 corresponding to “much improved,” 1 to “improved but retreatment needed,” 0 to “no change,” −1 to “worse,” and −2 to “much worse.”

Ordinal rank change scales were used by the patient to determine on a scale of 0 to 100, how much their attractiveness had changed as a result of their surgical journey, when compared with before the reconstructions. They were asked to rate their facial attractiveness on a scale of 0 to 100 prereconstruction and postreconstruction. The same scale and scoring system were also used by a panel of 10 peer-review plastic surgeons to give an ordinal rank change prereconstruction and postreconstruction for each of these patients with photographs shown. A positive score indicated an improvement in perceived attractiveness, and a negative score depicted a reduction in perceived facial attractiveness.

Statistical analysis was performed using Microsoft Excel with mean averages calculated for each scale and patient outcome for each PROM. A mean average was calculated from a summation of the peer-reviewer scores for ordinal rank change, and further averaged across all 35 patients in the case series to obtain 1 representative value. Written consent was provided, by which the patients agreed to the use and analysis of their data. The Declaration of Helsinki Ethical Principles for Medical Research Involving Human Subjects was followed in this study.

## RESULTS

A clinical case series was collated of 35 patients who had been through the 3D photography, MDT clinic, and 3D computer modeling integrated pathway. The average age of patients was 53.1 years (7-92 years). The ratio of male-to-female patients was 14:21. Patients were followed up for 1 year. The Table displays details on the patients’ demographics and the indications for their reconstructions. The Figure illustrates an example of a patient who presented postdebridement of a severe nasal squamous cell carcinoma (SCC) (A), who had 3D photographs taken from which measurements were made within the MDT clinic (B), and a computer model subsequently made prior to the next clinic meeting (C, D), illustrating the expected final reconstruction. The steps of this patient's reconstructive pathway involved a discussion with the patient with regard to the diagnosis prebiopsy, given the clinical impression of SCC, subsequent MDT clinic discussion with histology results, a plan for excision and histological checking of margins before reconstruction, then collation of old photographs to develop a 3D model to guide reconstruction. Each stage of the reconstructive process was discussed with the patient. This particular patient's reconstructive surgery was halted mid-stage to complete immunotherapy treatment as the patient developed SCC recurrence. The patient filled out the PROMs as this was the end of surgical treatment for now, but the aim is for them to return in the future for further reconstruction refinement once oncological therapy is complete.

**Figure.**
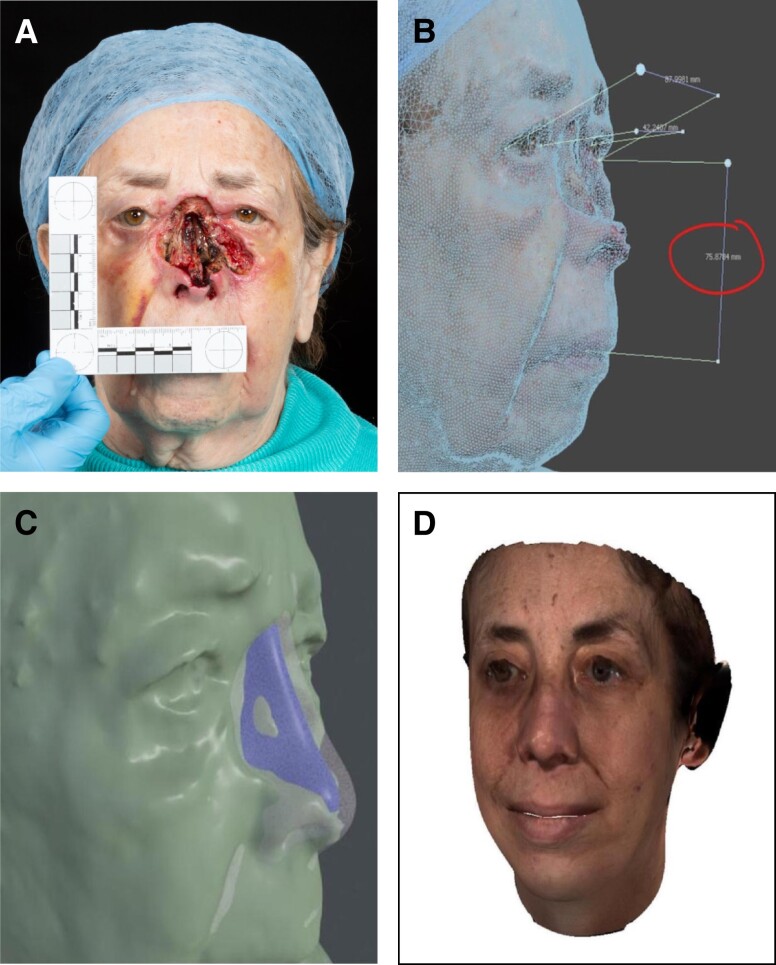
An 85-year-old female patient with severe nasal squamous cell carcinoma (SCC): (A) 2-month postdebridement of nasal SCC, before reconstruction; (B) 3D photography planning; (C) computer modeling of reconstruction; (D) soft tissue rendering of completed reconstruction.

**Table.** Demographics and Reconstruction Indications for Patients Involved in the MDT Clinic, 3D Photography, and 3D Modeling Pathway

Total no. of patients	35
Ratio of male:female patients	14:21
Mean age (years)	53.1
**Indication for reconstruction**	**No. of patients**
Squamous cell carcinoma	15
Basal cell carcinoma	5
Malignant melanoma	4
Hemifacial microsomia	6
Trauma	4
Congenital nasal atresia	1

3D, three-dimensional; MDT, multidisciplinary team.

The FACE-Q 10-item scale for satisfaction with facial appearance overall showed an average Rasch improvement of 80 (0-100). The FACE-Q 6-item scale for satisfaction with outcome showed an average Rasch improvement of 81 (0-100). The FACE-Q 6-item scale for satisfaction with decision showed an average Rasch improvement of 82 (0-100). The FACE-Q 7-item scale for aging appearance appraisal showed an average Rasch score of 21. The Self-Perception of Age measure showed an average perceived improvement in age-related facial appearance of −7.7 years postreconstruction when compared with prereconstruction. The FACE-Q 8-item scale for appearance-related psychological distress showed an average Rasch score of 9. The GAIS showed an average score of 1.7. The Ordinal Rank improvement on facial aesthetic appearance, as judged subjectively by the patient, was +17, on a scale with centile degradations. The Ordinal Rank improvement on facial aesthetic appearance, as judged by the panel of 10 reviewers, was +19.5, on the same scale.

## DISCUSSION

The results from this clinical case series involving 35 patients who underwent a 3D photography, MDT clinic, and 3D computer modeling integrated pathway are presented. The outcomes were measured using various scales and assessments related to satisfaction with facial appearance, decision making, aging appearance, psychological distress, aesthetic improvement, and subjective and objective judgments of facial aesthetic appearance (Appendices A, B).

### Satisfaction With Facial Appearance Overall and Outcome

The average Rasch improvement of 80 (0-100) on the FACE-Q 10-item scale for satisfaction with facial appearance overall suggests a significant enhancement in patients’ perception of their overall facial appearance after the intervention. This improvement indicates a positive impact on patient self-esteem and satisfaction.

Similarly, the FACE-Q 6-item scale for satisfaction with outcome yielded an average Rasch improvement of 81 (0-100). This outcome further supports the notion that patients were highly satisfied with the specific outcomes of the intervention, including parameters such as improved symmetry, proportions, or aesthetic features.

### Satisfaction With Decision

The average Rasch improvement of 82 (0-100) on the FACE-Q 6-item scale for satisfaction with decision indicates that patients were highly satisfied with the decision-making process involved in their treatment journey. This suggests that the integration of 3D photography and computer modeling in the decision-making process was effective in meeting patient expectations and preferences.

### Aging Appearance

The FACE-Q 7-item scale for aging appearance appraisal showed an average Rasch score of 21. This result implies that patients perceived a relatively youthful facial appearance after the reconstruction, indicating successful outcomes in addressing age-related changes and enhancing overall facial aesthetics.

Moreover, the self-perception of age measure revealed an average perceived improvement in age-related facial appearance of −7.7 years postreconstruction compared with prereconstruction. This finding suggests that patients felt their appearance became significantly younger following the intervention, contributing to their overall satisfaction, and is the result of paying attention to the aesthetic aspect of facial surgery.

### Appearance-Related Psychological Distress

The FACE-Q 8-item scale for appearance-related psychological distress yielded an average Rasch score of 9. This result indicates that patients experienced relatively low levels of psychological distress related to their facial appearance after the reconstruction. The intervention appears to have positively influenced patients’ psychological well-being and confidence throughout their reconstructive journey.

### Global Aesthetic Improvement

The GAIS showed an average score of 1.7, indicating an overall improvement in facial aesthetics according to the patients’ subjective evaluation. This result supports the previous findings of increased satisfaction and suggests that patients perceived a positive change in their appearance following the intervention.

### Ordinal Rank Improvement

The Ordinal Rank improvement on facial aesthetic appearance, as judged subjectively by the patients, was +17 on a scale with centile degradations. This implies a considerable improvement in patients’ perception of their facial aesthetics.

Additionally, when evaluated by a panel of 10 reviewers, the Ordinal Rank improvement on facial aesthetic appearance was +19.5, on the same scale. This result indicates that the improvement in facial aesthetics was also evident to external observers, validating the patients’ subjective assessments.

Facial aesthetic procedures are some of the most performed procedures in plastic surgery.^1^ In the last decade, health-care models have shifted, becoming less paternalistic and providing patients more autonomy. This, combined with the rapidly growing consumer population in the field of facial aesthetics, has been reflected in the expectations of patients undergoing facial aesthetic procedures and has meant that more patients are seeking reliable and valid data to drive informed decision making. Although conventional reporting of outcomes such as complication rates remains important, there has been a need for the reporting of other outcomes that can provide patients with additional valuable information that will enable them to make informed choices. PROMs are tools that can provide this information and are especially important in facial aesthetics in which clinicians strive to improve a patient's facial appearance as perceived by the patient.^2^

The lack of reliable and valid PROM tools, particularly in the field of facial aesthetics, led to the development of the FACE-Q, a PROM instrument that was specific to patients undergoing surgical and nonsurgical facial aesthetic procedures. The resulting FACE-Q tool consisted of at least 40 independent scales that included patient-reported outcome concepts deemed important to patients undergoing facial aesthetic procedures. Each scale consists of a series of statements to which responses are made on a 4-point Likert scale.^3^ The scales measured 4 main domains—Satisfaction with Facial Appearance, Health-Related Quality of Life, Negative Sequelae, and Satisfaction with Process of Care. To assess Satisfaction with Facial Appearance, scales for satisfaction with overall facial appearance and specific parts of the face were developed. This was done to accommodate patients undergoing different procedures on different parts of the face.^2^ The Health-Related Quality of Life concept examined the impact of the aesthetic procedure on the patient's psychological and social well-being. The Negative Sequelae domain encompassed any issues experienced by the patient during the recovery period such as bruising, swelling, as well as any adverse events. Satisfaction with Process of Care measured satisfaction with any information given to the patient, as well as with the care delivered by the medical team.^2^ All of these domains were assessed with FACE-Q scales in our study.

A modular approach to the FACE-Q has been adopted, whereby different subsets of scales can be used by clinicians depending on their purpose, ensuring that the scales used are relevant to a situation. For example, the FACE-Q Aesthetics module includes 24 scales that measure facial appearance, 10 scales that measure health-related quality of life, and 6 checklists that measure adverse effects.^1^ In contrast, the FACE-Q Skin Cancer module consists of 2 scales measuring facial appearance, 2 scales measuring quality of life, 1 scale measuring satisfaction with the information provided, and 2 checklists measuring sun protection behavior and adverse effects following treatment.^4^

Since its development, the FACE-Q has been used to assess outcomes across a range of facial aesthetic procedures, in both clinical practice and research, and its uptake has increased rapidly.^1^ It has been advocated as a reliable and valid PROM tool, capable of displaying high levels of responsiveness.^5^ A recent literature review that examined how the FACE-Q Aesthetics was being utilized by different researchers found that the tool provided valuable information to clinicians.^1^ For example, in rhinoplasty patients, the use of the FACE-Q enabled certain factors to be identified as potential predictors of success. These included age, race, and income, although reasons for this were outside the scope of the review. The authors also cited several studies that demonstrated the FACE-Q as able to detect small changes in satisfaction with facial appearance following peri-oral enhancement and facial contouring with fillers.^1^ Overall, the FACE-Q provides a standardized method through which patient-reported outcomes can be evaluated, allowing clinicians to gain a better understanding on what makes certain facial aesthetic procedures successful.

In the field of facial trauma reconstruction, Elegbede et al acknowledged the scarcity of patient-reported outcome tools and investigated the use of the FACE-Q in a cohort of patients with facial fractures that were sustained from trauma. The authors carried out a prospective study, in which 185 patients who had undergone primary reconstruction of their facial fractures were assessed 1 month postoperatively using 6 of the FACE-Q scales. The results were then compared with published scores from studies in the literature. All the scales evaluated were found to have good-to-excellent reliability. Their study supported the reliable use of the FACE-Q in facial trauma reconstruction patients, which may avoid the need to develop a separate PROM tool for facial trauma patients.^6^

Aside from the FACE-Q, other PROMs have also been used in facial aesthetic and reconstructive surgery. In rhinoplasty, several PROM instruments have been utilized, such as the Rhinoplasty Outcomes Evaluation (ROE-Q) and Sino-Nasal Outcome Test-22 (SNOT-22).^7^ The ROE-Q consists of a 6-point questionnaire answered by patients, with questions ranging from those asking about satisfaction with appearance to questions assessing nasal function.^8^ The SNOT-22 is a 22-point patient questionnaire that assesses problems such as nasal blockage, facial pain, and embarrassment, and the degree to which these problems affect patients. The reason for not using some of these scales in our study is the reduced amount of evidence supporting their use.

Another systematic review on PROMs for soft-tissue facial reconstructions found that the FACE-Q, Skin Cancer Index, Patient Outcome of Surgery-Head/Neck, and the Derriford Appearance Scale 59/24 (DAS 59/24) showed sufficient levels of psychometric and methodologic evidence to be used in future studies.^9^ The Patient Outcome of Surgery-Head/Neck consists of a 6-point presurgery and 9-point postsurgery questionnaires and has been used to evaluate skin lesions, both malignant and benign. The Derriford Appearance Scales are psychometric scales that have been used to measure psychological adjustment to appearance. The DAS 59/24 has been standardized on the clinical population of preoperative and postoperative plastic surgery patients as well as on the general population.^10^ It has been described as a highly sensitive tool that is capable of objectively measuring outcomes and providing valid and reliable data.^10^ The Skin Cancer Index is a tool that has been used in the assessment of nonmelanoma skin cancer. It contains 15 items, categorized into emotion, social, and appearance subscales, and with each item assessed on a 5-point Likert scale.^11^ It was shown to be a sensitive and responsive tool^12^ and a systematic review on PROMs on nonmelanoma skin cancer on the face identified that, at the time of the review, the Facial Skin Cancer Index was the only PROM specific to patients with nonmelanoma skin cancer on the face.^13^

In head and neck cancer, many PROM tools have been used in studies to assess the quality of life of patients, but many of these instruments have not been specific to patients with head and neck cancer and are instead directed to a particular symptom or issue.^14^ For example, when evaluating outcomes such as anxiety in patients with head and neck cancer, the Hospital Anxiety and Depression Scale (HADS-A) has often been used, which is a generic questionnaire used by clinicians to measure anxiety and depression in a general patient population. Having said this, some examples of PROM tools have been mentioned in the literature that are specific to patients with head and neck cancer. These include the EORTC Head and Neck Module (EORTC HN35)^15,16^ and a patient-reported questionnaire that includes statements relating to pain, swallowing, and speech.^16^ In 1 cross-sectional study in which the authors used the EORTC HN35 to assess the quality of life in patients with head and neck cancer after radiotherapy, they found the EORTC HN35 to be a well-validated and comprehensive tool. However, some limitations mentioned were that certain specific symptoms were not covered in the measure, such as deafness and otitis media.^16^ Other PROM tools that have been mentioned in the literature include FACT-HN, a 39-item questionnaire that has been reported to cover a full range of physical and emotional symptoms experienced by patients with head and neck cancer but may need to be supplemented with condition-specific measures depending on the objective of the clinician.^14,17^

The principles used in our study to develop the facial reconstruction integrated pathway can be extrapolated and used in all aspects of reconstructive surgery as well as aesthetic medicine in which photography and photo documentation are of paramount importance. Within the field of aesthetic medicine, complex aesthetic reconstructions such as rhinoplasty or those undertaken within consortium groups of aesthetic professionals, including surgeons and nonsurgical interventionalists would benefit from this system. Furthermore, there is an additional role for psychiatry specialists, given the prevalence of body dysmorphia in a standard population.^18^ The cost for 3D printing in this study was £15,000. Modeling was performed in-house and can be taught to team members through collaboration with university computer modeling departments as we did.

One limitation of our study was that there was no comparison group of patients who did not undergo the MDT clinic pathway. However, because this was a proof-of-concept study to provide evidence that integrating these systems leads to excellent outcomes, offering patients a lesser service would have been unethical. Another limitation of this study was that the outcomes of the study were mostly evaluated through subjective scoring systems. Although undoubtedly important, patient satisfaction with their facial appearance may not always accurately reflect how successful the procedure has been. To introduce some objectivity, facial appearance was also assessed by a panel of 10 plastic surgeons, but this was still based on subjective assessment from years of expertise.

## CONCLUSIONS

The results of this clinical case series highlight the positive impact of the integrated pathway involving 3D photography, MDT clinic, and 3D computer modeling on patient satisfaction with facial appearance, decision making, aging appearance, psychological distress, and overall aesthetic improvement. The findings indicate substantial improvements in various aspects, such as patient self-perception, subjective assessments, and external judgments of facial aesthetics. These results support the efficacy and potential of the integrated pathway in achieving desirable outcomes and enhancing patient well-being. Further research and larger studies could help validate these findings and provide additional evidence for the effectiveness of this integrated approach in facial reconstruction and aesthetic interventions, and can be expanded to other areas of reconstructive surgery and aesthetic medicine. Furthermore, in this study, further support for the use of PROMs in assessing surgical outcomes is provided.

## Supplementary Material

ojad082_Supplementary_Data
