# Supplementary material for: Patient-Reported Outcome Measures in a Facial Reconstruction Case Series Following the Implementation of an Integrated Craniofacial Multidisciplinary Team Clinic, Three-Dimensional Photography, and Computer Modeling
Source: Aesthet Surg J Open Forum. 2023 Sep 20;5:ojad082. doi: 10.1093/asjof/ojad082 (PMC10540727; doi:10.1093/asjof/ojad082)
Supplement: ojad082_Supplementary_Data [file ojad082_supplementary_data.zip › 23-0085_Appendix B.docx]

**
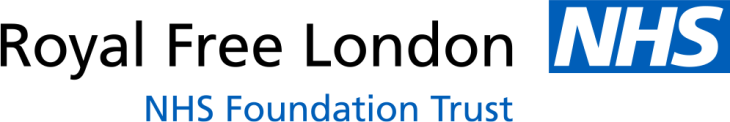
Ordinal Rank Change Review**

Please rank the following patients on their facial attractiveness on a Likert scale of 0(completely unattractive) to 100(perfectly attractive), pre-op and post-op. Please write the number (0-100) in the table for each patient.


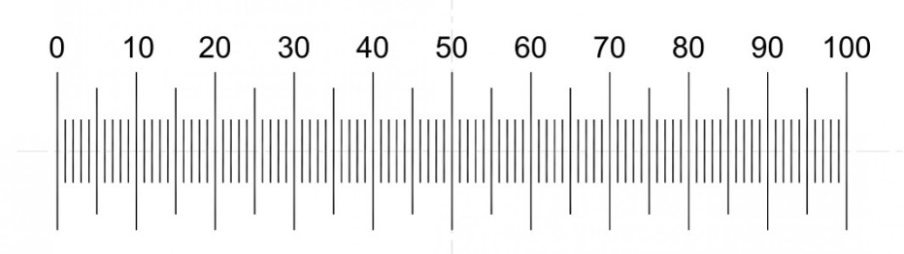


| Patient  Number | Pre-Op Attractiveness | Post-Op Attractiveness | Ordinal Rank Change +/- |
| --- | --- | --- | --- |
| 1 |  |  |  |
| 2 |  |  |  |
| 3 |  |  |  |
| 4 |  |  |  |
| 5 |  |  |  |
| 6 |  |  |  |
| 7 |  |  |  |
| 8 |  |  |  |
| 9 |  |  |  |
| 10 |  |  |  |
| 11 |  |  |  |
| 12 |  |  |  |
| 13 |  |  |  |
| 14 |  |  |  |
| 15 |  |  |  |
| 16 |  |  |  |
| 17 |  |  |  |
| 18 |  |  |  |
| 19 |  |  |  |
| 20 |  |  |  |
| 21 |  |  |  |
| 22 |  |  |  |
| 23 |  |  |  |
| 24 |  |  |  |
| 25 |  |  |  |
| 26 |  |  |  |
| 27 |  |  |  |
| 28 |  |  |  |
| 29 |  |  |  |
| 30 |  |  |  |
| 31 |  |  |  |
| 32 |  |  |  |
| 33 |  |  |  |
| 34 |  |  |  |
| 35 |  |  |  |
