# Supplementary material for: Patient-Reported Outcome Measures in a Facial Reconstruction Case Series Following the Implementation of an Integrated Craniofacial Multidisciplinary Team Clinic, Three-Dimensional Photography, and Computer Modeling
Source: Aesthet Surg J Open Forum. 2023 Sep 20;5:ojad082. doi: 10.1093/asjof/ojad082 (PMC10540727; doi:10.1093/asjof/ojad082)
Supplement: ojad082_Supplementary_Data [file ojad082_supplementary_data.zip › 23-0085_Appendix A.docx]

**
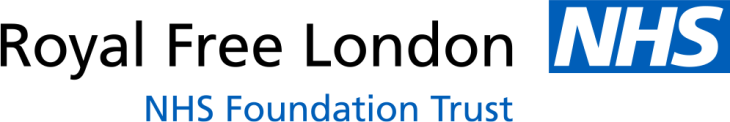
Patient Satisfaction Questionnaire**

1. **FACE-Q 10-item Satisfaction with Facial Appearance Overall**

For each statement circle only one answer about your satisfaction with your facial appearance. Please indicate how satisfied you are from very dissatisfied to very satisfied for the following 10 statements;

| **Facial appearance criteria** | **Very dissatisfied** | **Somewhat dissatisfied** | **Somewhat satisfied** | **Very satisfied** |
| --- | --- | --- | --- | --- |
| 1. How symmetrical your face looks? | 1 | 2 | 3 | 4 |
| 1. How balanced your face looks? | 1 | 2 | 3 | 4 |
| 1. How well-proportioned your face is? | 1 | 2 | 3 | 4 |
| 1. How your face looks at the end of day? | 1 | 2 | 3 | 4 |
| 1. How fresh your face looks? | 1 | 2 | 3 | 4 |
| 1. How rested your face looks? | 1 | 2 | 3 | 4 |
| 1. How your face looks in profile? | 1 | 2 | 3 | 4 |
| 1. How your face looks in photos? | 1 | 2 | 3 | 4 |
| 1. How your face looks when you first wake up? | 1 | 2 | 3 | 4 |
| 1. How your face looks under bright lights? | 1 | 2 | 3 | 4 |

1. **Satisfaction with Outcome**

For each statement circle only one answer about your satisfaction with the outcome of your procedure. Please indicate how much you disagree or agree with the following 6 statements;

| **Facial appearance criteria** | **Definitely disagree** | **Somewhat disagree** | **Somewhat agree** | **Definitely agree** |
| --- | --- | --- | --- | --- |
| 1. I am pleased with the result | 1 | 2 | 3 | 4 |
| 1. The result turned out great | 1 | 2 | 3 | 4 |
| 1. The result was just as I expected | 1 | 2 | 3 | 4 |
| 1. I am surprised at how good I look in the mirror | 1 | 2 | 3 | 4 |
| 1. The result is fantastic | 1 | 2 | 3 | 4 |
| 1. The result is miraculous | 1 | 2 | 3 | 4 |

1. **Satisfaction with Decision**

For each statement circle only one answer about your satisfaction with the decision to have your procedure. Please indicate how much you disagree or agree with the following 6 statements;

| **Decision criteria** | **Definitely disagree** | **Somewhat disagree** | **Somewhat agree** | **Definitely agree** |
| --- | --- | --- | --- | --- |
| 1. It was worth the time and effort | 1 | 2 | 3 | 4 |
| 1. It was money well spent | 1 | 2 | 3 | 4 |
| 1. It was just what I wanted | 1 | 2 | 3 | 4 |
| 1. It was just what I needed | 1 | 2 | 3 | 4 |
| 1. It made me look how I want to look | 1 | 2 | 3 | 4 |
| 1. It changed my life for the better | 1 | 2 | 3 | 4 |

1. **FACE-Q Aging Appearance Appraisal**

For each statement circle only one answer about how you rate your facial appearance now, after your reconstruction. Please indicate how much you agree or disagree with each of the following 7 statements;

| **Facial appearance criteria** | **Definitely disagree** | **Somewhat disagree** | **Somewhat agree** | **Definitely agree** |
| --- | --- | --- | --- | --- |
| 1. I look so old that I don’t recognise myself | 1 | 2 | 3 | 4 |
| 1. When I look in the mirror I don’t look like myself | 1 | 2 | 3 | 4 |
| 1. I am bothered by how old I look | 1 | 2 | 3 | 4 |
| 1. I look older than I want to look | 1 | 2 | 3 | 4 |
| 1. I am worried by how old I am starting to look | 1 | 2 | 3 | 4 |
| 1. In recent photos I look older than I want to | 1 | 2 | 3 | 4 |
| 1. When I see my reflection I am reminded of how old I look | 1 | 2 | 3 | 4 |

1. **Self Perception of Age**

Please write how old you feel you look, and your actual age, in years. Please also indicate how many ears younger or older you think you look compared to your actual age.

| **Actual Age (years)** | **How old I feel I look (years)** |
| --- | --- |
|  |  |


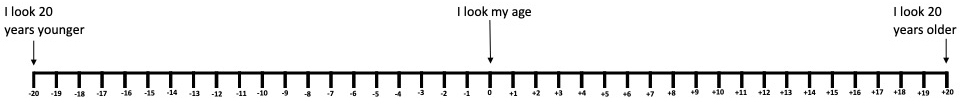


1. **FACE-Q Appearance related Psychological Distress**

For each statement circle only one answer about your psychological distress at your facial appearance. Please indicate how much you agree or disagree with each of the following 8 statements;

| **Facial appearance criteria** | **Definitely disagree** | **Somewhat disagree** | **Somewhat agree** | **Definitely agree** |
| --- | --- | --- | --- | --- |
| 1. I feel unhappy about how I look | 1 | 2 | 3 | 4 |
| 1. I feel stressed about how I look | 1 | 2 | 3 | 4 |
| 1. I feel down about how I look | 1 | 2 | 3 | 4 |
| 1. I feel anxious when people look at me | 1 | 2 | 3 | 4 |
| 1. I worry that I don’t look normal | 1 | 2 | 3 | 4 |
| 1. I worry that I am ugly | 1 | 2 | 3 | 4 |
| 1. I tend to avoid being around people | 1 | 2 | 3 | 4 |
| 1. I have little interest in doing things | 1 | 2 | 3 | 4 |

1. **Global Aesthetic Improvement Scale (GAIS)**

Please indicate on the following 5 point scale how you rate your global improvement in aesthetic appearance of your face, with;

2 = much improved (marked improvement in appearance),

1 = improved (improvement in appearance but a touch-up or re-treatment is indicated),

0 = no change (appearance essentially the same as original condition),

−1 = worse (appearance worse than original condition),

−2 = much worse (appearance much worse than original condition).


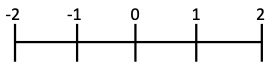


1. **Ordinal Rank Change (Patient)**

Please rate your opinion on the appearance of your facial attractiveness as a change from a scale from 0(completely unattractive) to 100(perfectly attractive), pre-op and post-op. Please write the number (0-100) in the box below the scale

Pre-op:


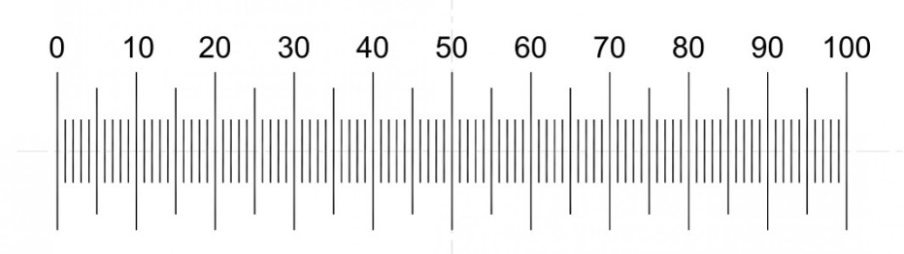


Post-op:


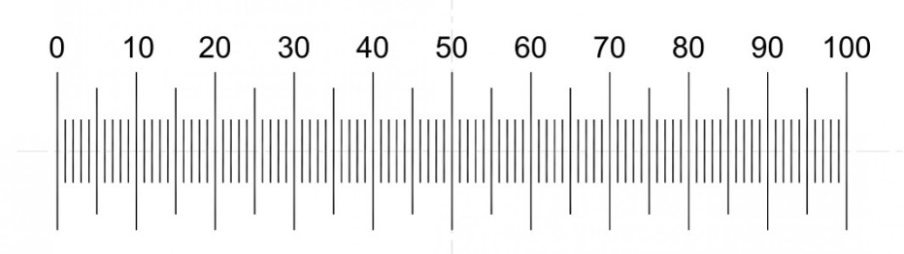


1. **Ordinal Rank Change (Peer reviewer)**

Please rate your opinion on the appearance of this patients facial attractiveness as a change from a scale from 0 to 100, pre-op and post-op
